# Estimating policy-relevant health effects of ambient heat exposures using spatially contiguous reanalysis data

**DOI:** 10.1186/s12940-019-0467-5

**Published:** 2019-04-18

**Authors:** Temilayo E. Adeyeye, Tabassum Z. Insaf, Mohammad Z. Al-Hamdan, Seema G. Nayak, Neil Stuart, Stephen DiRienzo, William L. Crosson

**Affiliations:** 1 Bureau of Environmental and Occupational Epidemiology, New York State Department of Health, Albany, NY USA; 2 Universities Space Research Association, NASA Marshall Space Flight Center, Huntsville, AL USA; 3 Department of Epidemiology and Biostatistics, School of Public Health, University at Albany, State University of New York, Rensselaer, NY USA; 4 National Oceanic and Atmospheric Administration/ National Weather Service, Albany, NY USA

**Keywords:** Extreme heat, Evidence-based public health, Heat advisory, Reanalysis

## Abstract

**Background:**

Regional National Weather Service (NWS) heat advisory criteria in New York State (NYS) were based on frequency of heat events estimated by sparse monitoring data. These may not accurately reflect temperatures at which specific health risks occur in large geographic regions. The objectives of the study were to use spatially resolved temperature data to characterize health risks related to summertime heat exposure and estimate the temperatures at which excessive risk of heat-related adverse health occurs in NYS. We also evaluated the need to adjust current heat advisory threshold and messaging based on threshold temperatures of multiple health outcomes.

**Methods:**

We assessed the effect of multi-day lag exposure for maximum near-surface air temperature (T_max)_ and maximum Heat Index derived from the gridded National Land Data Assimilation System (NLDAS) reanalysis dataset on emergency department (ED) visits/ hospitalizations for heat stress, dehydration, acute kidney failure (AKF) and cardiovascular diseases (CVD) using a case-crossover analysis during summers of 2008–2012. We assessed effect modification using interaction terms and stratified analysis. Thresholds were estimated using piecewise spline regression.

**Results:**

We observed an increased risk of heat stress (Risk ratio (RR) = 1.366, 95% confidence interval (CI): 1.347, 1.386) and dehydration (RR = 1.024, 95% CI: 1.021, 1.028) for every 1 °C increase in T_max_ on the day of exposure. The highest risk for AKF (RR = 1.017, 95% CI: 1.014, 1.021) and CVD (RR = 1.001, 95% CI: 1.000, 1.002) were at lag 1 and 4 respectively. The increased risk of heat-health effects persists up to 6 days. Rural areas of NYS are at as high a risk of heat-health effects as urban areas. Heat-health risks start increasing at temperatures much lower than the current NWS criteria.

**Conclusion:**

Reanalysis data provide refined exposure-response functions for health research, in areas with sparse monitor observations. Based on this research, rural areas in NYS had similar risk for health effects of heat. Heat advisories in New York City (NYC) had been reviewed and lowered previously. As such, the current NWS heat advisory threshold was lowered for the upstate region of New York and surrounding areas. Enhanced outreach materials were also developed and disseminated to local health departments and the public.

**Electronic supplementary material:**

The online version of this article (10.1186/s12940-019-0467-5) contains supplementary material, which is available to authorized users.

## Introduction

While residents of New York State (NYS) have largely experienced moderate temperatures during the summers, the frequency and intensity of hot days and heat events have been increasing and are projected to continue to rise over the next several decades [1]. Many studies of health risks associated with extreme ambient heat exposures have been conducted in urban areas and rely on assigning exposure based on distance to a single monitor [2, 3]. Air monitors are sparsely located and tend to be concentrated in urban areas. In situ data therefore have limited utility in studying heat effects over large areas that may include suburban or rural areas which lack air monitoring stations [4]. The North American Land Data Assimilation System (NLDAS) which combines remote sensing, in situ and model data to provide fine scale temperature metrics at the near surface level [5, 6], makes it possible to assign more precise data statewide [7]. The NLDAS has been used to calculate relative temperature metrics [8]. The application of spatially resolved finer scale reanalysis data to estimate public health effects associated with extreme heat is a recent development for NYS climate research. This spatially contiguous temperature dataset allows for the quantification of areal heterogeneity in health effects related to heat.

Furthermore, the fine scale temperature data allow us to determine heat-health thresholds for excessive risk which can better inform initiatives to protect health during heat events, such as the National Weather Service (NWS) heat alert system. Regional NWS forecast offices issue excessive heat alerts (advisories, watches, and warnings) based on the maximum heat index forecasts over 24–72 h [9]. Heat index is a metric that combines temperature and humidity to estimate conditions most likely to affect human health. An advisory is issued when the heat index is forecast to exceed 37.8 °C (100 °F) for 2 h or more for upstate NYS regions [10]. Appropriate heat warnings may prevent morbidity due to heat exposures. However, current temperature thresholds for heat advisories and warnings in upstate NYS were established over 20 years ago and were not based on heat-health associations. Previous studies have also proposed the consideration of health events and diagnoses for identifying threshold temperatures [11, 12]. The NLDAS reanalysis dataset provides the opportunity to conduct heat-health analysis for all regions of NYS and reassess the criteria for heat advisories, so they are more relevant to temperatures experienced in NYS during the summer.

The aims of this study were to characterize morbidity risk related to summertime heat exposure using residential address-based health records for emergency department (ED) visits / hospitalizations (May through September, 2008–2012) in NYS. We incorporated reanalysis data (12-km NLDAS products) to define exposure-response functions for regions where observed data from air-monitors are unavailable. In addition, we evaluated current heat advisory criteria in the region and established heat-health thresholds for excessive risks of morbidity in order to inform health policy.

## Methods

### Study design and population

We applied case-crossover analysis to assess the association between temperature and counts of daily hospital admissions and ED visits for heat stress, dehydration, acute kidney failure (AKF), and cardiovascular diseases (CVD) in NYS in the summers of 2008 through 2012.

### Health outcomes

Daily summer hospital admissions and ED visits (May – September) in NYS were acquired from the New York State Department of Health’s Statewide Planning and Research Cooperative System (SPARCS) inpatient and outpatient datasets. SPARCS contains billing and medical abstract information for each hospital inpatient stay and outpatient (ambulatory surgery, ED, and outpatient services) visit in the state; except those at Federal and Veterans Administration hospitals [13]. Outpatient visits and ambulatory surgeries were excluded from the CVD analysis as these were unlikely to arise from acute heat exposures. The New York State Department of Health (NYSDOH) Institutional Review Board and Data Protection Review Board approvals were obtained to access individually identifiable information such as address, date of birth, and date of hospital visit. For each outcome, we combined both hospital admissions and ED visits for data analyses due to small sample size. In total, four health outcomes were assessed: heat stress (*n* = 8703), dehydration (*n* = 59,828), AKF (*n* = 50,008) and CVD (*n* = 845,927).  The following international classifications of diseases (ICD, revisions 9) were used for heat stress (ICD-9, 992.0–992.9; E900.0, E900.9), dehydration (ICD-9, 276.51), AKF (ICD-9, 584.5–584.9), and CVD (ICD-9, 390–398, 401–405, 410–417, 420–438, 440–448, 451–459). For acute conditions such as heat stress and dehydration, any recurrences of ED visits and/or hospitalizations within a 1-week period were excluded, with recurrences after that period considered as new events. While for chronic conditions such as AKF and CVD, recurrences within a 28-day period were excluded. Residential addresses obtained from hospital admission records were geocoded at street-level and assigned longitude and latitude coordinates, using NYS Street Address Mapping (SAM) [14], MapMarker® [15], and ArcGIS® [16].

### Air temperatures

#### Reanalysis dataset

The NLDAS dataset integrates a large quantity of observation- based and model reanalysis data executed at a grid surface with ∼14 km (1/8th degree) resolution over North America. The non-precipitation land-surface forcing fields for NLDAS are originally derived from the analysis fields of the National Centers for Environmental Prediction (NCEP) North American Regional Reanalysis (NARR). NARR analysis fields have a 32-km spatial resolution and 3-hourly temporal frequency. The NARR fields that are used to generate NLDAS meteorological fields are spatially interpolated to the finer resolution of the NLDAS 1/8th-degree grid and then temporally disaggregated to the NLDAS hourly frequency [5]. The NLDAS hourly data used in this study were acquired as part of the mission of NASA’s Earth Science Division and archived and distributed by the Goddard Earth Sciences (GES) Data and Information Services Center (DISC). This hourly data slightly differs from the temperature data typically reported from an air monitor station which has only minimum and maximum temperature capabilities.

**Fig. 1:**
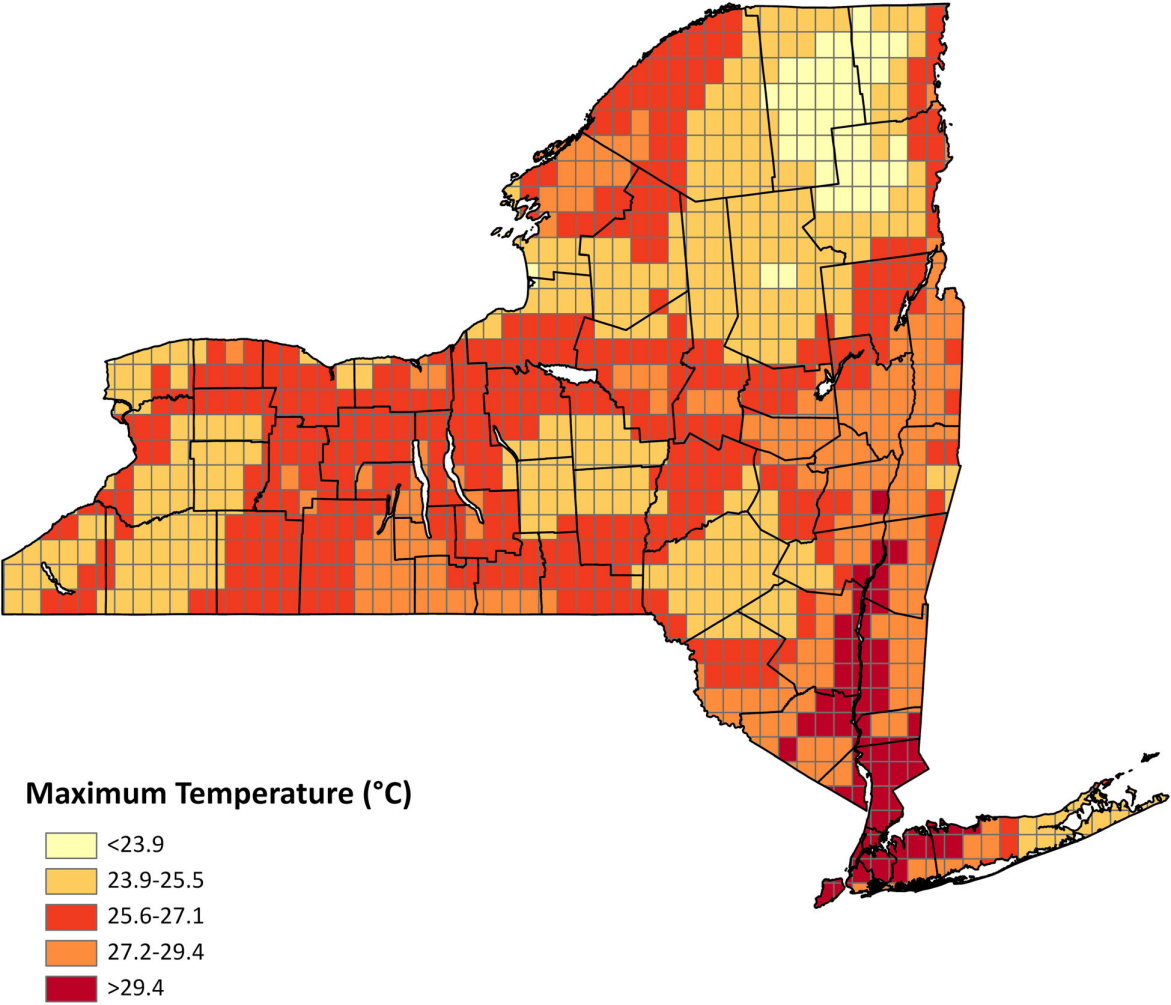
North American Land Data Assimilation System (NLDAS) 12 KM grid displaying maximum temperature (°C) in New York State for July 21, 2010

From the NLDAS hourly data, we derived daily maximum temperature (T_max_), minimum temperature (T_min_), and mean temperature (T_mean_) data (all in °F), and a daily maximum heat index (maxHI) product, which reflect the combined effects of heat and humidity [8]. For a description of how the maximum heat index was calculated, please refer to Additional file 1: Appendix A. The NLDAS data consists of 103,936 grid cells covering the entire U.S., excluding Alaska and Hawaii. For this study, 1040 grids covering NYS were used (Fig. 1). In the absence of information on the actual place of exposure, the temperature metrics in this gridded dataset were assigned based on the grid that included the patient’s residential address. This dataset was then spatially merged with the datasets containing fine particulate matter (PM_2.5_) and ozone estimates using the geographic coordinates of the patients’ residences. Lagged exposure values were created for all models to assess the immediate (on admission day, i.e. lag 0) and delayed (exposure up to 7 days before admission i.e. lag 1–7 respectively) effects of the temperature metrics on the health outcomes. The temperature effect at different lags were modeled separately. Effect of cumulative exposures were assessed using moving averages for multiple lags. We also used a distributed lag structure where multiple lags were included simultaneously in the model.

#### Monitor dataset

Automated Surface Observing System (ASOS) units are automated sensor suites that are designed to serve meteorological and aviation observing needs [17]. ASOS systems generate reports at hourly intervals and serve as a primary climatological observing network in the United States. We extracted daily temperature data from 32 ASOS units stationed across NYS. We used 100-mile and 20-mile buffer ranges around the 32 ASOS stations, to spatially join the heat stress cases (*n* = 8799) during the study period (2008–2012) with ASOS stations closest to each observation.

### Air pollution data

Daily estimates of PM_2.5_ and ozone were derived using data from the Environmental Protection Agency’s (EPA) downscaler (DS) model [18]. The DS model fuses output from a gridded atmospheric model with point air pollution measurements. It combines air quality monitoring and modeling data to provide finer-scale predictions of air pollutant levels at local and community levels [19]. We categorized air pollutant estimates as low and high exposures using the 25th percentile and 75th percentile of the data distribution respectively.

### Potential effect modifiers

Urban areas are typically warmer than rural areas due to urban heat island effects. However, we were interested in evaluating whether rural areas had a consequently lower risk of heat-health outcomes. The rural-urban commuting area (RUCA) codes which classify U.S. census tracts using measures of population density, urbanization, and daily commuting were used to categorize the rural-urban areas of NYS [20]. The most recent RUCA codes are based on data from the 2010 decennial census and the 2006–2010 American Community Survey. New York City (NYC) is by far the most densely populated, urbanized area in NYS. It also has a more diverse population with socio-demographic characteristics that may not represent other urban populations in NYS. In addition, NYC is serviced by its own local health department. Therefore, it was deemed appropriate to conduct a separate analysis for that area. The rest of NYS was divided into urban and rural census tracts based on RUCA codes.

We also assessed whether effects of heat vary by individual and socioeconomic characteristics. The hospitalization/ED data provided information on age, sex, race and ethnicity. Age was categorized into 6 categories. Race and ethnicity information in the medical records was classified as Hispanic, White Non-Hispanic, Black/Non-Hispanic and Other. To evaluate whether existing chronic illness modified effects of heat among cases with a primary diagnosis code of heat stress and dehydration, we flagged cases with pre-existing diabetes, chronic kidney disease, heart failure, other heart disease, hypertension, chronic liver disease, acute renal disease, acute myocardial infarction and atherosclerosis in “Other Diagnoses” fields.

### Statistical methods

We conducted a case-crossover analysis using a semi-symmetric bidirectional, time-stratified design to assess the effect of summertime temperature on hospitalizations and ED visits in NYS. This method compares the temperature metrics on the day of hospital admission /visit (case/exposure day) with the temperature metrics, before and/or after (control period), within the same pre-specified stratum window of time, when the subject is not hospitalized or in the ED [21]. Since each case serves as his or her own control on all measured and unmeasured subject non-time-varying characteristics, confounding factors such as individual demographic and social factors have been adjusted by design [22]. A one-month stratum window of exposure was used to compare cases with the control (referent) period of ±7, ±14, or ± 21 days from case day within the same month, thereby providing up to four control dates per case. The season and day of the week variations were controlled for by restricting the referents to the same day of the week, month and year as the index day [23]. We controlled for time varying variables such as PM_2.5_ and ozone in the model.

The initial analysis was conducted in °Fahrenheit to conform to NWS criteria for issuance of heat warning. In this manuscript, all changes in risk based on temperature are reported in S.I. units (°C) to allow for comparison with previous research. Although analyses were carried out for maximum temperature, minimum temperature, mean temperature and maximum heat index, we focus on maximum heat index which is widely used by the NWS and heat-health researchers in the United States.

### Heat-health associations and effect modification

We initially calculated risk ratios for a 1 °C change in temperature metric using conditional logistic regression analysis for each health outcome. Effect modification for age, sex, race/ethnicity, rural/urban areas, and month of exposure were determined a priori based on literature review and assessed using multiplicative interaction terms. The joint effect of ozone and PM_2.5_ on heat-health were also evaluated using interaction terms. There has been some debate about the adjustment for air pollution when assessing risk of health effects of heat [24, 25]. Therefore, we present results with and without adjustment for ozone and PM_2.5_. We assessed rural-urban differences in heat health associations. We assessed effects separately by NYC, non-NYC urban and non-NYC rural. Finally, we evaluated effect modification for effects on heat on heat stress and dehydration in individuals with pre-existing comorbidities using a stratified analysis. We conducted a supplementary analysis using monitor data linked to health observations using 20- and 100-mile buffer zones to compare with estimates derived from the spatially contiguous reanalysis data. We repeated the case-crossover analysis using monitor data and compared results with those from reanalysis dataset.

### Heat-health threshold analysis

To establish regional heat advisory thresholds that are relevant for heat-health associations in the region, we used a piecewise linear spline regression to assess the shape of the temperature-outcome association. Knots defining slope changes were sequentially selected at 5-degree intervals. We calculated three separate trigger points as suggested by recent research [11, 26]. The minimum risk temperature (MRT) for heat stress was determined as the lowest temperature at which the health outcome was observed during the study period. For all other health outcomes, the MRT was defined as the lowest temperature above which a consistent increase in the relative risk was observed. The excess risk temperature (ERT) was defined as the lowest temperature above the MRT at which the lower bound of the 95% confidence interval of relative risk of a particular health outcome was greater than 1. We assessed the risk ratios at the existing NWS criteria of 37.8 °C (100 °F) and at the proposed criteria of 35 °C (95 °F).

All statistical analyses were carried out using SAS™ statistical software Version 9.4. (SAS Institute Inc., Cary, NC, USA) and R. Geographic Information System (GIS) analysis was carried out using SAS and ArcGIS® [16].

## Results

There were 964,466 cases included in this study (Table 1). The population was predominantly White non-Hispanic and about 53% were female (Table 1).

**Table 1:** Distribution of Hospital Visits and Admissions in New York State (May – September 2008 – 2012)

Variables	Heat Stress	Dehydration	Acute Kidney Failure	Cardiovascular Illnesses^a^
	Cases (%)	Cases (%)	Cases (%)	Cases (%)
Number of cases	8,703 (23.29)	59,828 (23.70)	50,008 (23.69)	827,051 (23.49)
Inpatient hospitalizations	1,338	28,938	48,943	614,062
ED cases	7,365	30,890	1,065	212,989
Control days	28,664 (76.71)	192,571 (76.30)	161,089 (76.31)	2,693,862 (76.51)
Case Month				
May	755 (8.68)	11,198 (18.72)	8,898 (17.79)	176,141 (21.30)
June	2,243 (25.77)	12,575 (21.02)	10,324 (20.64)	164,132 (19.85)
July	4,009 (46.06)	14,414 (24.09)	11,264 (22.52)	163,281 (19.74)
August	1,278 (14.68)	11,776 (19.68)	10,352 (20.70)	162,873 (19.69)
September	418 (4.80)	9,865 (16.49)	9,170 (18.34)	160,624 (19.42)
Age, years				
≤ 4	124 (1.42)	7,078 (11.83)	44 (0.09)	1,946 (0.24)
5 – 24	2,344 (26.93)	8,856 (14.80)	600 (1.20)	15,619 (1.89)
25 – 44	2,310 (26.54)	7,941 (13.27)	2,660 (5.32)	77,147 (9.33)
45 – 64	2,180 (25.05)	11,202 (18.72)	12,320 (24.64)	265,822 (32.14)
65 – 84	1,353 (15.55)	15,918 (26.61)	23,508 (47.01)	347,501 (42.02)
≥ 85	392 (4.50)	8,833 (14.76)	10,876 (21.75)	119,016 (14.39)
Sex				
Male	5,106 (58.67)	26,063 (43.56)	25,891 (51.77)	421,710 (50.99)
Female	3,597 (41.33)	33,764 (56.44)	24,117 (48.23)	405,331 (49.01)
Missing	-	1 (0.00)	-	10 (0.00)
Race/Ethnicity				
White, Non-Hispanic	5,288 (60.76)	37,486 (62.66)	30,016 (60.02)	487,602 (58.96)
Hispanic	1,144 (13.14)	7,283 (12.17)	4,928 (9.85)	88,164 (10.66)
Black, Non-Hispanic	1,482 (17.03)	9,193 (15.37)	10,522 (21.04)	160,396 (19.39)
Other	707 (8.12)	5,525 (9.23)	4,371 (8.74)	87,130 (10.54)
Missing/Unknown	82 (0.94)	341 (0.57)	171 (0.34)	3,759 (0.45)
NYC	2,964 (34.06)	21,662 (36.21)	21,665 (43.32)	361,219 (43.68)
Rest of NYS	5,739 (65.94)	38,166 (63.79)	28,343 (56.68)	465,832 (56.32)

^a^excludes observations that were coded as outpatient (clinic) services or Ambulatory services

**Fig. 2:**
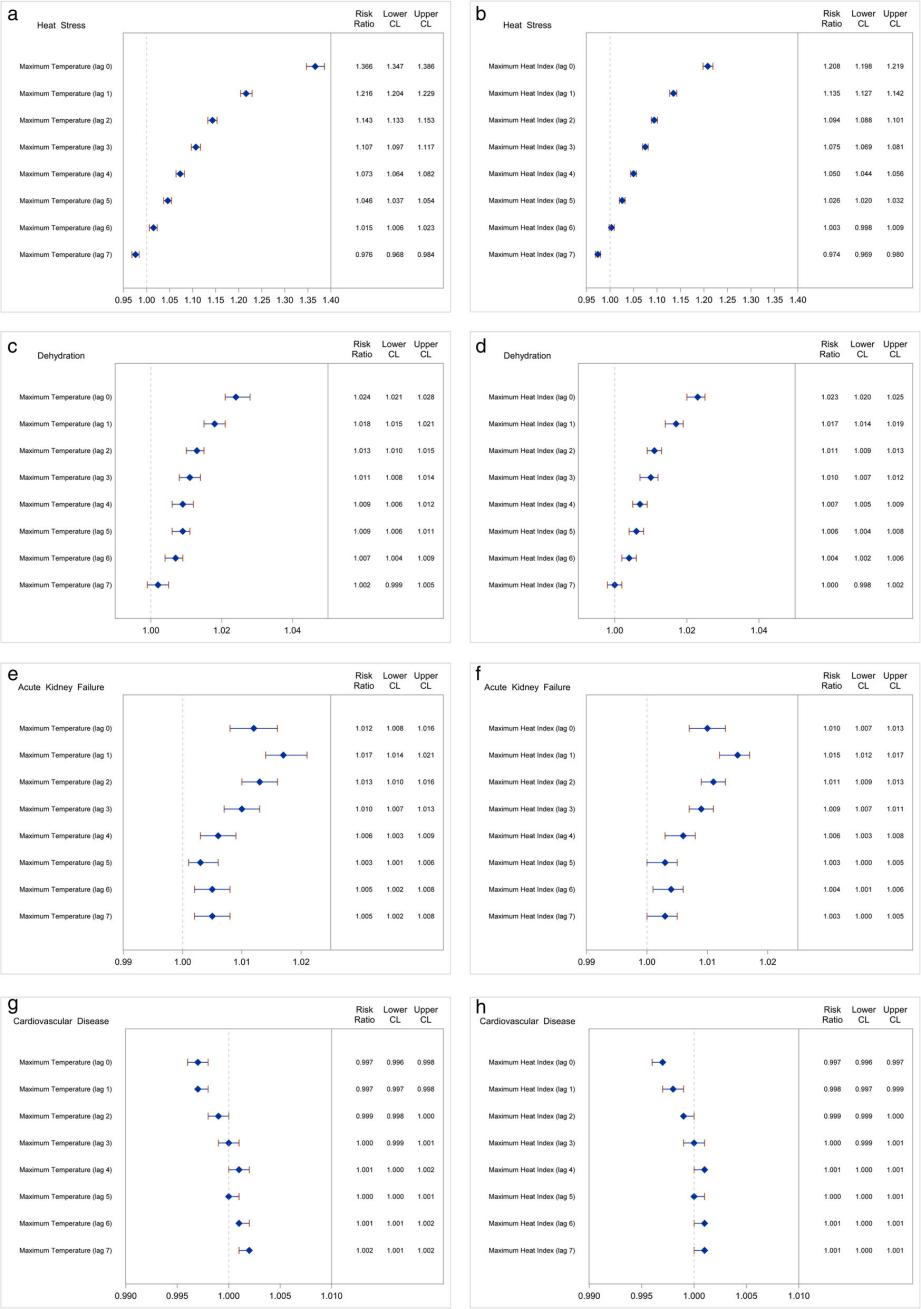
Risk of hospitalization/emergency room visit associated with a 1°C change in lagged temperatures in New York State (2008–2012)

There were significant increases in the risk of hospital visit/ admission for heat stress & dehydration across all temperature indicators (Fig. 2). We present the risk ratios between a 1 °C change in T_max_ and maxHI for all health outcomes assessed in this study using the single lag models. Results from the distributed lag models were in agreement with the single lag models and are presented in Additional file 1: Tables S2–S5. We present the risk from a combined analysis of ED and hospitalization visits. Results from separate analysis of ED and inpatient hospitalizations yielded similar results and are presented in Additional file 1: Table S1. The largest estimates between T_max_ and heat stress hospital admissions/visits were on the same-day of admission (lag 0). There was an increased risk of heat stress for every 1 °C increase in maximum temperature (Risk ratio (RR) = 1.366, 95% confidence interval (CI): 1.347, 1.386). The lag effects for heat stress lasted for 6 days. Similarly, the highest magnitude of risk for dehydration was for same-day exposure (RR = 1.024, 95% CI: 1.021, 1.028). The risk for dehydration was highest at lag 0, with decreasing magnitude of association as lag day increases. There were significant increases in the risk of hospital visits/admissions for AKF at all lags (Fig. 2). However, the highest magnitude of association between temperature and an increased risk of AKF hospital visits/admissions was at lag 1. For every 1 °C increase in T_max_, there was an increased risk of ED visit/ hospitalization for AKF at lag 1 (RR = 1.017, 95% CI: 1.014, 1.021) and CVD at lag 4 (RR = 1.001, 95% CI: 1.000, 1.002). The probability of cardiovascular disease ED visits/ hospitalizations on the same-day of exposure (lag 0) was significantly decreased for all temperature indicators suggesting a lag-effect of temperature. We present cumulative effects of temperature on all health outcomes in Additional file 1: Figure S1.

### Joint effects and effect modification

We tested if the associations between temperature and the health outcomes were modified by variables such as age, sex, race/ethnicity, ozone, PM_2.5_, rural/urban areas, and month of exposure by adding a product term between temperature and each modifier into the model (Table 2 and Additional file 1: Tables S6–S8). The highest risk was found among the oldest age groups for heat stress, but differences were not statistically significant. Females were less likely to be hospitalized or visit the emergency room for dehydration-related health issues. Although the highest risk of heat stress occurred in the summer months of June, July and August, the cooler shoulder months of May and September also show elevated risk of heat stress. There is a complex relationship between exposures to particulate matter, ozone and high temperature. The risk of heat stress with 1 °C change in temperature was highest on days with low ozone and high PM_2.5_ and lowest on days with high ozone and low particulate matter exposures. The risk of heat on heat stress, dehydration and AKF were lowered when adjusted for particulate matter and ozone. The effects of one-degree change in temperature on ED visits/admissions for heat stress (Table 2) were similar in both rural and urban areas in NYS. We present results of effect modification for other health outcomes in Additional file 1: Tables S6–S8. Participants with chronic conditions had an overall lower risk of heat stress and dehydration than those who did not have comorbidities (Additional file 1: Figure S2).

**Table 2:** Association Between a 1°C Change in Maximum Temperature and Heat Stress (May – September 2008 –2012)

Demographic Variables and Subgroups^a^	Risk Ratio (95% CI)
Age, years^b^	
4 or younger	1.318 (1.217, 1.427)
5 – 24	1.339 (1.310, 1.369)
25 – 44	1.372 (1.341, 1.403)
45 – 64	1.377 (1.345, 1.409)
65 – 84	1.386 (1.346, 1.427)
85 or older	1.400 (1.330, 1.474)
Sex^b^	
Male	1.363 (1.340, 1.386)
Female	1.370 (1.345, 1.396)
Race/Ethnicity^b^	
White, Non-Hispanic	1.374 (1.351, 1.397)
Black, Non-Hispanic	1.338 (1.304, 1.373)
Hispanic	1.371 (1.330, 1.414)
Other	1.365 (1.315 1.416)
Month^b^	
May	1.373 (1.329, 1.418)
June	1.370 (1.341, 1.400)
July	1.361 (1.335, 1.388)
August	1.388 (1.340, 1.439)
September	1.326 (1.270, 1.384)
NYC only^b^	1.371 (1.338, 1.405)
Rest of NYS^b^	
Rural NYS	1.373 (1.302, 1.448)
Urban NYS excluding NYC	1.362 (1.337, 1.388)
Low Ozone/ Low PM_2.5_^c^	1.356 (1.334, 1.379)
High Ozone/ Low PM_2.5_^c^	1.287 (1.263, 1.312)
Low Ozone/ High PM_2.5_^c^	1.434 (1.405, 1.463)
High Ozone/ High PM_2.5_^c^	1.361 (1.342, 1.381)
Unadjusted	1.433 (1.418, 1.448)
Adjusted for PM_2.5_ only	1.367 (1.348, 1.385)
Adjusted for Ozone only	1.400 (1.382, 1.419)
Adjusted for PM_2.5_ & Ozone	1.366 (1.347, 1.386)

^a^*Abbreviations*: *RR* Risk Ratio, *CI* confidence interval

^b^Adjusted for ozone and PM_2.5'_

^c^ Low ozone= 33.95ppb (25^th^ percentile), high ozone = 52.56ppb (75^th^ percentile); low PM_2.5_ = 6.26 μg/m^3^ (25^th^ percentile), high PM_2.5_ = 13.06 μg/m^3^ (75^th^ percentile)

### Heat-health threshold analysis

**Fig. 3:**
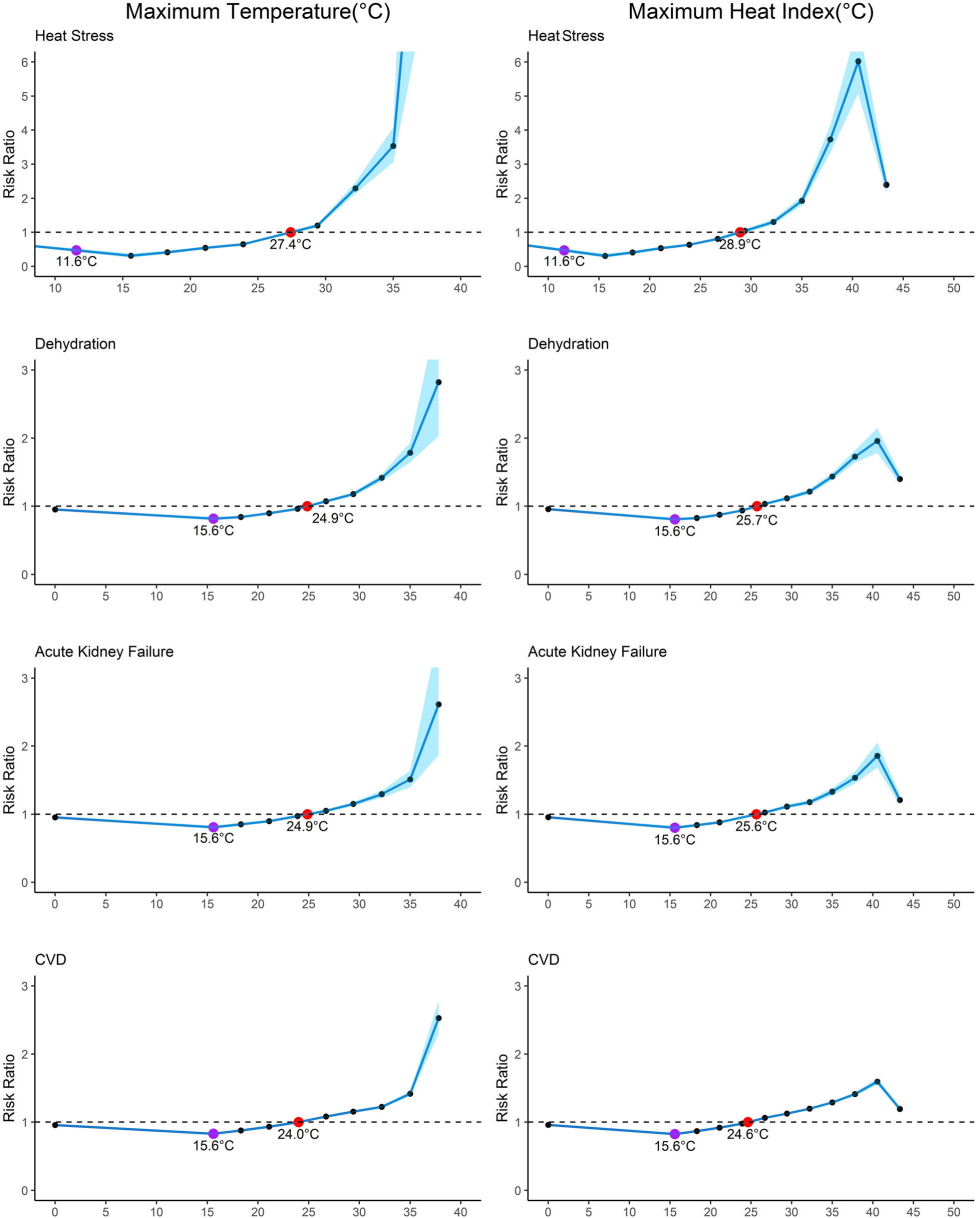
Modeled relationship between risk ratio of hospitalization/ ED visit four health outcome and same-day temperature metrics (heat stress & dehydration)/ lagged temperature metrics (acute kidney failure & CVD) during the summers of 2008 – 2012 in New York State. Specific points labeled on the curve identify the minimum risk temperature (MRT, purple) and the excess risk temperature (ERT, red), representing different conceptualizations of trigger points for intervention

We evaluated multiple trigger points for health effects of heat exposures (Fig. 3). The MRT for each heat-health association was 10–15 °C lower than its ERT. For maximum temperatures, the risk for heat-health outcomes showed an exponential pattern beyond the MRTs for all health outcomes. The heat index charts show similar trends until a heat index of 40 °C (104 °F) with a leveling or decline in risk at extremely high temperatures. For heat stress, the ERT was defined at 28.8 °C (83.8 °F) while the ERTs were between 24 and 26 °C (75.2–78.8 °F) for all other health outcomes. At the pre-existing NWS threshold of 37.8 °C (100 °F), the risk ratio for heat stress was 3.727 while the risk ratio for other health outcomes ranged from 1.727 for dehydration, 1.534 for AKF and 1.412 for CVD. In contrast, at a reduced heat advisory criterion of 35 °C (95 °F), the risk ratio for heat stress is 1.927 and ranges from 1.436 for dehydration, 1.329 for AKF and 1.290 for CVD.

### Comparison with monitor data

Monitor data were especially sparse in rural areas. Additional file 1: Figures S3 – S5 show ASOS stations, gridded data, and the 20- and 100- mile buffers. As is evident, there is considerable spatial variability in the gridded data within the 20- and 100- mile buffer zones. Using a 20-mile buffer, 11.7% of the case population had to be dropped from the analysis because they were too far from an air monitor to be assigned a value. Using a 100-mile buffer, all of the case population were included in the analysis but there was a considerable overlap in buffer zones. We therefore assigned the temperature values based on the monitor closest to the individual’s address. As expected, the smaller sample size resulted in wider confidence intervals in estimates for health effects in rural areas using a 20-mile buffer. In addition, the risk ratios were attenuated for both rural and urban areas using the 100 mile buffer (Additional file 1: Tables S9–S12 ).

## Discussion

We examined the association between ambient summer temperatures and ED visits/hospital admissions for multiple health outcomes using fine-scaled environmental data over a broad geographic region. There has been a recent interest in using spatially resolved data to estimate health effects of heat in wider geographical regions [4, 27]. The use of a spatially contiguous exposure fields allows estimation of heat effects in small cities, rural and suburban areas that lack dense air monitoring networks. In addition, the case-crossover design minimized bias due to inter-individual variation and consequent uncontrolled confounding in epidemiological studies of heat exposures [22].

The goals of this study were to 1) assess the effect of heat on health using fine-scale reanalysis data and 2) use our findings to better inform policies that protect the health of NYS residents during periods of extreme heat. Our results show significant risks of heat stress, dehydration, and AKF from lags 0 through 6. We found that effects of heat can start at moderately high values of Heat Index much below the current thresholds for NWS advisories in the area. In its most recent directive for the Eastern United States region, the NWS has recognized that emergency department visits due to heat illness may begin well before NWS thresholds are met, thus forecasters have been directed to focus on issuing long-lead watches along with early, enhanced safety messaging via Public Information Statements and social media [10].

We observed a positive relationship between summer high temperatures and acute occurrences of heat stress and dehydration with lagged effect. The same-day exposure effects that we observed for heat stress and dehydration are expected due to the direct relationship between heat and these health outcomes; and are consistent with literature [28]. We found that significant risk of heat stress persisted up to 6 days after exposure. We found an elevated risk of acute renal illness from lag 0, with the highest risk at lag 1 for renal illness. We observed only a modest increase in risk of cardiovascular illness at lags 4–6. These findings are consistent with other studies of health effects in temperate climate [29, 30].

We also explored the potential effect modification by individual characteristics such as age, sex, race, ethnicity, rural/urban areas, and exposure month for all health outcomes. People aged 25–44 years were more susceptible to dehydration and heat stress compared to those between ages 5 and 24 years. The increased risk in the 25–44 group may be elevated because of subjects in this category have a higher likelihood of being exposed to heat through occupational and/or recreational exposures. Higher risk among older adults between 65 and 84 years old may be due to existing comorbid conditions that may make them more susceptible to heat stress [31].

The risk of dehydration in the transition months (May and September) is lower in magnitude than the hotter months of June, July and August. This may be due to the infrequency of events during this period. However, since the risk is still statistically significant, it suggests that when heat events occur in transition months, people are less prepared and likely to experience adverse health outcomes. There have been inconsistent findings for heat-health risk by gender [32–35]. In this study, we did not find significant differences between the risk of heat stress among females and males. But females were less likely to have dehydration-related ED visit/admissions compared to males. This is consistent with results from the Center for Disease Control and Preventions’ Environmental Public Health Tracking Program survey, where males were found to be more affected by adverse heat-related conditions than females [36]. These differences may be due to sex differences in sweat threshold, sweat rate, morphology (more muscle and less fat in men) and different levels of hormones regulating fluid balance [32]. They could also reflect the tendency for men to engage in more outdoor work than women, especially in the construction and agricultural industries [33–35].

Furthermore, we explored the spatial distribution of effects of temperature on heat-health outcomes in NYS. As expected, the metropolitan NYC area had the highest risk; likely due to its geographic location and the Urban Heat Island effect that results from building materials that retain heat and increased anthropogenic heat production [37, 38]. Rural areas in the region are perceived to be at lower risk due to a temperate climate and uncommon heat events. However, risk ratios for other urban centers and rural areas in NYS were comparable. We surmise that with increasing temperatures, a lack of climate mitigation measures such as access to home air-conditioners and public cooling centers, may lead to an increase in susceptibility to heat morbidity in these regions [37, 39].

Although we would expect that those with chronic health conditions may be at a higher risk, we observed an overall lower risk of hospitalization/ED visit for heat stress and dehydration in this group. It has been suggested that those with chronic long-standing conditions are more likely to avoid outdoor exposures during unpleasant or extreme weather, thus resulting in a null or negative correlation with temperature [24, 40]. A sub-analysis of comorbid conditions among those who visited the hospital for heat stress and dehydration during the study period supported this hypothesis (Additional file 1: Figure S2).

Heat warning systems utilize myriad approaches to characterize threshold temperatures for adverse effects of heat and form an important part of regional heat mitigation and climate adaptation policies [11, 41, 42]. Recognizing that the Excessive Heat Warning / Heat Advisory criteria should be based on regional climate variability and the effect of excessive heat on the local population, the NWS encourages regional offices to work with health departments and develop criteria based on scientific evidence derived from local data [43]. The NYSDOH has therefore worked closely with the local weather service offices to develop a recommendation to lower the heat advisory thresholds in our region based on results of this study. Other efforts in the northeastern US including NYC where criteria have been changed based on the predicted number of deaths attributable to forecast heat events were based on monitoring data from mostly urban centers [43, 44]. Prior to this study, the heat advisory threshold for the upstate NY region and surrounding areas was 37.8 °C (100 °F) or more for two consecutive hours. However, our research findings have shown that this criteria for issuing heat advisories may not be sufficiently protective for public health. The ERT for heat stress morbidity using the maximum heat index is 28.8 °C (83 °F) and the MRTs for other health outcomes in the study were between 24 and 26 °C (75.2–78.8 °F). We recommended that a conservative heat advisory threshold of 35 °C (95 °F) be considered for the general public, as that would capture a high proportion of heat events likely to result in significant morbidity, while avoiding warning fatigue if frequent advisories were issued at lower temperatures. Based on research findings and recommendations, four NWS offices (Albany, NY; Binghamton, NY; Buffalo, NY; and Burlington, VT) changed their heat advisory criteria for New York, effective on or about June 1st, 2018 to 35 °C (95 °F) or more for two consecutive hours. Lower advisory criteria for high risk populations such as outdoor workers, student athletes and school children are either already in place or being considered in the region [45–47]. The NWS heat-advisory threshold for NYC had been lowered previously [43]. We also recommend that heat awareness messaging be initiated early in the summer as our research suggests that significant morbidity risks can occur at temperatures lower than the NWS heat advisory thresholds.

Current public health interventions to increase awareness of heat exposure symptoms and provide advice for staying cool focus on public outreach to urban populations. Local health departments in rural areas are at the forefront of combating climate effects but are challenged with competing priorities and lack of tools and resources [48]. Using spatially resolved climate and health data from this project, NYSDOH has developed tools and infographics for each county in NYS to aid climate health surveillance and mitigation efforts [49]. Findings from this project have also been included in extreme heat messaging issued by the NYSDOH and disseminated through public websites and social media.

As with any epidemiologic analysis of administrative datasets, there are some limitations of our study that should be considered when interpreting results. We used hospital administrative data to identify individuals with the health outcomes assessed. Residential addresses were used to assign exposure temperatures in lieu of information on personal activities including exposure to indoor temperatures; and may not reflect the point of exposure, thereby leading to exposure misclassification if the exposure occurred elsewhere. However, the use of case-crossover design and same-day of the week selection for control days help in minimizing the bias introduced. We also do not know how behavioral adaptation relates to exposure such as access to air conditioning and cooling centers. Factors of the built environment that increase temperature such as housing type and socio-economic factors were not available. Occupational exposures were also not accounted for in this analysis. In urban areas, where land cover is predominantly non-vegetated, differences between the Land Surface Temperature (LST) and air temperature can be large. LST is the radiative skin temperature of the surface and is usually higher than air temperature. Our use of NLDAS air temperature data obviates this complication. NLDAS temperature data give estimates of the ambient temperature, derived from air monitor, remote sensing, and weather model data. The NLDAS air temperature compares relatively well with measured monitor data, where available [50]. Systematic differences exist in the association between temperature and health outcome based on time and type of temperature observations; and may consequently translate into differences in estimated temperature-health effects [51]. Although the spatial resolution of the NLDAS 1/8th-degree grid temperature data provides a more accurate measure of exposure than sparse monitoring networks, the urban scale features such as urban heat island effects may not be well-captured. Other studies comparing health effects using spatially resolved models with those estimated using monitoring data have demonstrated a better model fit probably due to better estimation of temperature variability in small areas [4]. Our study only estimated heat risks on morbidity. Previous studies evaluating heat risks of mortality have found lower risk ratios and higher threshold temperatures for mortality [11, 27].

## Conclusions

In this project, we demonstrate the application of spatially resolved climate data in informing climate policy for a large region comprising urban and rural areas. Future efforts will be needed to assess health impacts on mortality. Our efforts have led to policy changes around definition and public health information on regional heat advisories. We continue to monitor how a change in heat advisory threshold will impact public health effects of heat in the region.

## Additional file

Additional file 1: Supplementary document. (DOCX 1220 kb)

## Abbreviations

ARF: Acute Renal Failure
CI: Confidence Interval
CVD: Cardiovascular Disease
DISC: Data and Information Services Center
DS: Downscaler
ED: Emergency Department
EPA: Environmental Protection Agency
ERT: Excess Risk Temperature
GES: Goddard Earth Sciences
GIS: Geographic Information System
ICD: International Classifications of Diseases
LST: Land Surface Temperature
maxHI: Maximum Heat Index
MRT: Minimum Risk Temperature
NARR: North American Regional Reanalysis
NCEP: National Centers for Environmental Prediction
NLDAS: North American Land Data Assimilation System
NWS: National Weather Service
NYS: New York State
NYSDOH: New York State Department of Health
PM_2.5_: Fine particulate matter
RR: Risk Ratio
RUCA: Rural Urban Commuting Area
SAM: Street Address Mapping
SPARCS: Statewide Planning and Research Cooperative System
T_max_: Maximum Temperature
