# Supplementary material for: Estimating policy-relevant health effects of ambient heat exposures using spatially contiguous reanalysis data
Source: Environ Health. 2019 Apr 18;18:35. doi: 10.1186/s12940-019-0467-5 (PMC6471902; doi:10.1186/s12940-019-0467-5)
Supplement: Supplementary file 1 — Supplementary document. (DOCX 1220 kb) [file 12940_2019_467_MOESM1_ESM.docx]

**Supplementary Document**

Heat Index Calculations.

Heat Index (HI) arose from Steadman (1979) who referred to a quantity that combined temperature and humidity into an index of comfort both as ‘apparent temperature’ and a ‘temperature-humidity index’. Rothfusz (1990) performed a multiple regression analysis using Steadman’s (1979) tabular results to obtain an expression for what has become known as the ‘Heat Index’, but is sometimes still referred to as apparent temperature, given (in degrees F) by the following:

HI = -42.379 + 2.049015T + 10.143331R - 0.2247554TR - 6.83783🞄10^-3^T^2^ - 5.481717🞄10^-2^R^2^ + 1.22874🞄10^-3^T^2^R + 8.5282🞄10^-4^TR^2^ - 1.99🞄10^-6^T^2^R^2^ (1)

where T = air temperature (^o^F) and R = relative humidity. R is calculated from Iribarne and Godson [1981] as:

$R=\frac{q}{\left[ \frac{0.622\cdot{10}^{\left[ 23.547- \frac{2937.4}{\mathrm{TK}} -4.9283\cdot\log_{10}\mathrm{TK} \right]}}{p} \right]}$ (2)
where q = specific humidity (kg/kg), p = atmospheric pressure (mb), and TK = air temperature (Kelvins). NLDAS provides hourly near-surface (2 m above ground) specific humidity and atmospheric pressure data used in (2). Figure A tabulates HI values from (1) over a wide range of temperature and relative humidity conditions. Yellow, orange and red shading indicates values of 90, 105 and 130, respectively.

Figure A. Heat Index (^o^F) as a function of air temperature (^o^F) and relative humidity (%).

**References**

Iribarne JV, Godson WL: *Atmospheric Thermodynamics.* 2nd ed. Dordrecht, Holland: D. Reidel;

1981.

Rothfusz LP: The Heat Index 'Equation' (or, More Than You Ever Wanted to Know About Heat

Index). Scientific Services Division (NWS Southern Region Headquarters), SR 90-23, 1 July

1990.

Steadman, R.G., 1979. The assessment of sultriness. Part I: A temperature-humidity index based

on human physiology and clothing science. *J. Appl. Meteor.,* 18, 861-873.

| **Table S1.** Emergency Department and Inpatient Hospitalizations (Combined & Separate) Analysis | | | |
| --- | --- | --- | --- |
| Maximum Temperature | Heat Stress (All)^a^ | Heat Stress (ED cases)^a^ | Heat Stress (non-ED cases)^a^ |
| Lag 0 | 1.366 (1.347, 1.386) | 1.366 (1.345, 1.388) | 1.373 (1.324, 1.424) |
| Lag 1 | 1.216 (1.204, 1.229) | 1.206 (1.192, 1.219) | 1.275 (1.241, 1.310) |
| Lag 2 | 1.143 (1.133, 1.153) | 1.132 (1.121, 1.143) | 1.203 (1.175, 1.231) |
| Lag 3 | 1.107 (1.097, 1.117) | 1.096 (1.085, 1.106) | 1.176 (1.149, 1.205) |
| Lag 4 | 1.073 (1.064, 1.082) | 1.067 (1.058, 1.077) | 1.109 (1.084, 1.135) |
| Lag 5 | 1.046 (1.037, 1.054) | 1.045 (1.036, 1.055) | 1.049 (1.027, 1.071) |
| Lag 6 | 1.015 (1.006, 1.023) | 1.014 (1.005, 1.023) | 1.017 (0.997, 1.038) |
| Lag 7 | 0.976 (0.968, 0.984) | 0.978 (0.970, 0.987) | 0.965 (0.946, 0.985) |

^a^Adjusted for ozone and PM_2.5_

| **Table S2.** Risk Ratio (95% CI) for Lag Effect of Maximum Temperature on Health Outcomes in Separate Models | | | | |
| --- | --- | --- | --- | --- |
| Maximum Temperature Lag | Heat Stress | Dehydration | Acute Kidney Failure | CVD |
| 0 | 1.366 (1.347, 1.386) | 1.024 (1.021, 1.028) | 1.012 (1.008, 1.016) | 0.997 (0.996, 0.998) |
| 1 | 1.216 (1.204, 1.229) | 1.018 (1.015, 1.021) | 1.017 (1.014, 1.021) | 0.997 (0.997, 0.998) |
| 2 | 1.143 (1.133, 1.153) | 1.013 (1.010, 1.015) | 1.013 (1.010, 1.016) | 0.999 (0.998, 1.000) |
| 3 | 1.107 (1.097, 1.117) | 1.011 (1.008, 1.014) | 1.010 (1.007, 1.013) | 1.000 (0.999, 1.001) |
| 4 | 1.073 (1.064, 1.082) | 1.009 (1.006, 1.012) | 1.006 (1.003, 1.009) | 1.001 (1.000, 1.002) |
| 5 | 1.046 (1.037, 1.054) | 1.009 (1.006, 1.011) | 1.003 (1.001, 1.006) | 1.000 (1.000, 1.001) |
| 6 | 1.015 (1.006, 1.023) | 1.007 (1.004, 1.009) | 1.005 (1.002, 1.008) | 1.001 (1.001, 1.002) |
| 7 | 0.976 (0.968, 0.984) | 1.002 (0.999, 1.005) | 1.005 (1.002, 1.008) | 1.002 (1.001, 1.002) |

| **Table S3.** Risk Ratio (95% CI) for Lag Effect of Maximum Heat Index on Health Outcomes in Separate Models | | | | |
| --- | --- | --- | --- | --- |
| Maximum Heat Index Lag | Heat Stress | Dehydration | Acute Kidney Failure | CVD |
| 0 | 1.208 (1.198, 1.219) | 1.023 (1.020, 1.025) | 1.010 (1.007, 1.013) | 0.997 (0.996, 0.997) |
| 1 | 1.135 (1.127, 1.142) | 1.017 (1.014, 1.019) | 1.015 (1.012, 1.017) | 0.998 (0.997, 0.999) |
| 2 | 1.094 (1.088, 1.101) | 1.011 (1.009, 1.013) | 1.011 (1.009, 1.013) | 0.999 (0.999, 1.000) |
| 3 | 1.075 (1.069, 1.081) | 1.010 (1.007, 1.012) | 1.009 (1.007, 1.011) | 1.000 (0.999, 1.001) |
| 4 | 1.050 (1.044, 1.056) | 1.007 (1.005, 1.009) | 1.006 (1.003, 1.008) | 1.001 (1.000, 1.001) |
| 5 | 1.026 (1.020, 1.032) | 1.006 (1.004, 1.008) | 1.003 (1.000, 1.005) | 1.000 (1.000, 1.001) |
| 6 | 1.003 (0.998, 1.009) | 1.004 (1.002, 1.006) | 1.004 (1.001, 1.006) | 1.001 (1.000, 1.001) |
| 7 | 0.974 (0.969, 0.980) | 1.000 (0.998, 1.002) | 1.003 (1.000, 1.005) | 1.001 (1.000, 1.001) |

| **Table S4.** Risk Ratio (95% CI) for Lag Effect of Maximum Temperature on Health Outcomes in Same Model | | | | |
| --- | --- | --- | --- | --- |
| Maximum Temperature Lag | Heat Stress | Dehydration | Acute Kidney Failure | CVD |
| 0 | 1.283 (1.262, 1.305) | 1.017 (1.012, 1.021) | 1.001 (0.996, 1.006) | 0.998 (0.997, 0.999) |
| 1 | 1.064 (1.048, 1.081) | 1.007 (1.003, 1.012) | 1.015 (1.010, 1.020) | 0.998 (0.997, 0.999) |
| 2 | 1.040 (1.024, 1.056) | 1.001 (0.997, 1.005) | 1.001 (0.996, 1.005) | 1.000 (0.999, 1.002) |
| 3 | 1.000 (0.984, 1.016) | 1.003 (0.999, 1.007) | 1.006 (1.001, 1.010) | 1.000 (0.998, 1.001) |
| 4 | 1.012 (0.997, 1.028) | 0.999 (0.995, 1.004) | 0.999 (0.994, 1.003) | 1.002 (1.001, 1.003) |
| 5 | 0.997 (0.982, 1.012) | 1.003 (0.999, 1.008) | 0.997 (0.993. 1.002) | 0.999 (0.998, 1.000) |
| 6 | 1.000 (0.985, 1.014) | 1.002 (0.998, 1.006) | 1.002 (0.998, 1.007) | 1.001 (1.000, 1.002) |
| 7 | 0.962 (0.951, 0.973) | 1.000 (0.996, 1.003) | 1.003 (1.000, 1.007) | 1.001 (1.000, 1.002) |

| **Table S5.** Risk Ratio (95% CI) for Lag Effect of Maximum Heat Index on Health Outcomes in Same Model | | | | |
| --- | --- | --- | --- | --- |
| Maximum Heat Index Lag | Heat Stress | Dehydration | Acute Kidney Failure | CVD |
| 0 | 1.155 (1.143, 1.167) | 1.015 (1.012, 1.018) | 1.000 (0.996, 1.004) | 0.997 (0.996, 0.998) |
| 1 | 1.049 (1.039, 1.059) | 1.009 (1.006, 1.012) | 1.012 (1.009, 1.016) | 0.999 (0.998, 1.000) |
| 2 | 1.025 (1.015, 1.035) | 0.999 (0.996, 1.003) | 1.001 (0.997, 1.005) | 1.000 (0.999, 1.001) |
| 3 | 1.004 (0.993, 1.014) | 1.003 (1.000, 1.007) | 1.005 (1.001, 1.008) | 1.000 (0.999, 1.001) |
| 4 | 1.006 (0.996, 1.017) | 0.999 (0.996, 1.003) | 1.000 (0.997, 1.004) | 1.002 (1.001, 1.003) |
| 5 | 0.998 (0.988, 1.009) | 1.002 (0.999, 1.006) | 0.998 (0.994, 1.001) | 0.999 (0.998, 0.999) |
| 6 | 1.001 (0.991, 1.012) | 1.001 (0.998, 1.004) | 1.001 (0.998, 1.005) | 1.001 (1.000, 1.002) |
| 7 | 0.982 (0.974, 0.990) | 1.000 (0.997, 1.002) | 1.003 (1.000, 1.006) | 1.000 (1.000, 1.001) |

**Figure S1.** Cumulative Risk Ratios (95% Confidence Intervals) for All Health Outcomes


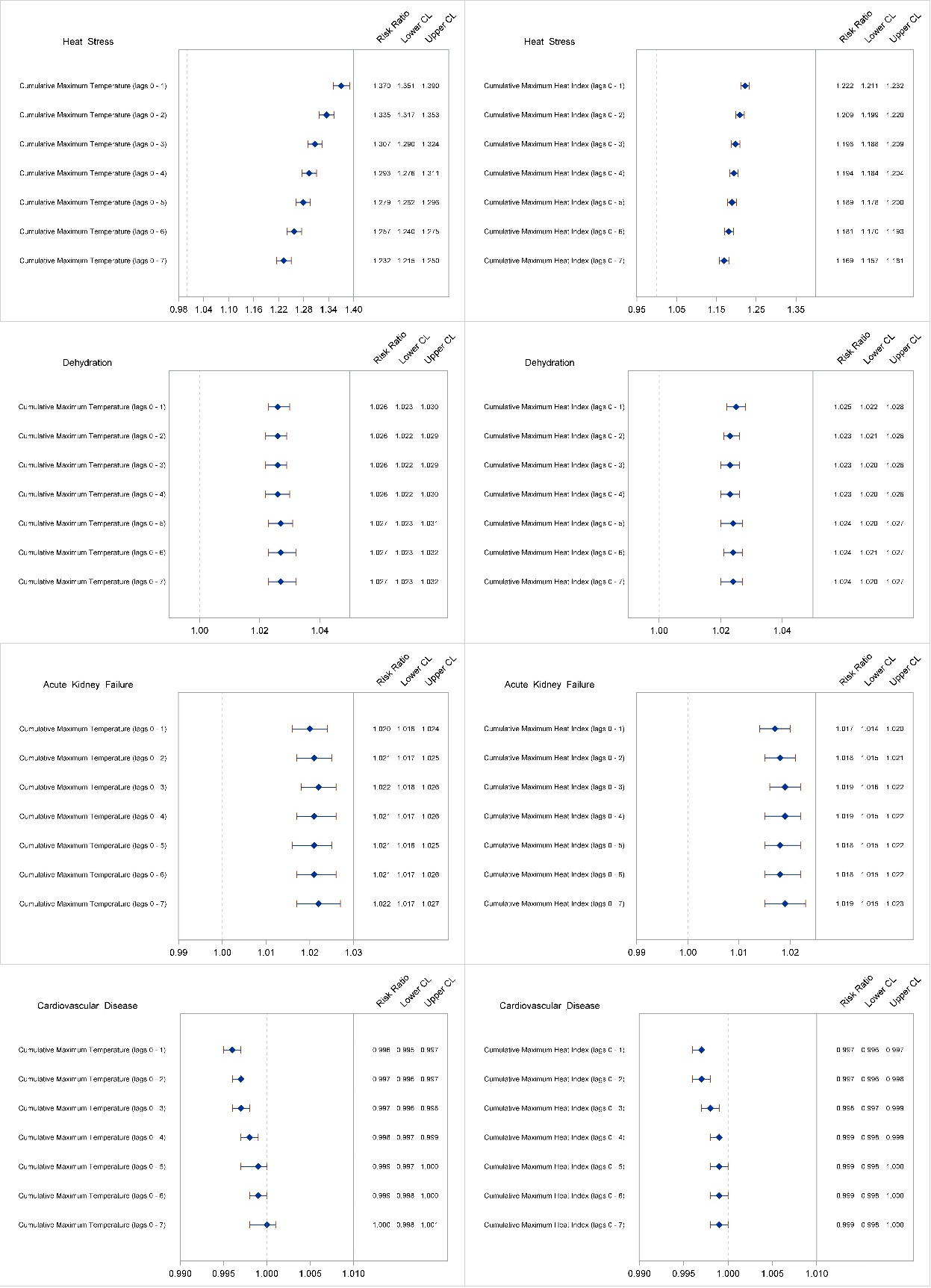


| Table S6. Association Between a 1°C Change in Maximum Temperature and Dehydration (May – September 2008 –2012) | |
| --- | --- |
| Demographic Variables and Subgroups^a^ | Risk Ratio (95% CI) |
| Age, years^b^ |  |
| 4 or younger | 1.005 (0.997, 1.012) |
| 5 – 24 | 1.029 (1.021, 1.036) |
| 25 – 44 | 1.039 (1.032, 1.047) |
| 45 – 64 | 1.030 (1.023, 1.037) |
| 65 – 84 | 1.021 (1.016, 1.027) |
| 85 or older | 1.022 (1.014, 1.029) |
| Sex^b^ |  |
| Male | 1.030 (1.025, 1.035) |
| Female | 1.020 (1.016, 1.024) |
| Race^b^ |  |
| White, Non-Hispanic | 1.024 (1.020, 1.028) |
| Black, Non-Hispanic | 1.031 (1.024, 1.038) |
| Hispanic | 1.020 (1.012, 1.028) |
| Other | 1.019 (1.010, 1.028) |
| Month^b^ |  |
| May | 1.019 (1.014, 1.024) |
| June | 1.035 (1.028, 1.041) |
| July | 1.043 (1.035, 1.052) |
| August | 1.038 (1.029, 1.047) |
| September | 1.014 (1.007, 1.020) |
|  |  |
| NYC^b^ | 1.032 (1.026, 1.038) |
| Rest of NYS^b^ |  |
| Rural NYS | 1.027 (1.014, 1.042) |
| Urban NYS excluding NYC | 1.019 (1.014, 1.024) |
| Air Pollutants |  |
| Low Ozone/ Low PM_2.5_^c^ | 1.022 (1.018, 1.025) |
| High Ozone/ Low PM_2.5_^c^ | 1.026 (1.021, 1.031) |
| Low Ozone/ High PM_2.5_^c^ | 1.029 (1.024, 1.035) |
| High Ozone/ High PM_2.5_^c^ | 1.034 (1.029, 1.038) |
| Mediation by PM_2.5_ & Ozone |  |
| Unadjusted | 1.033 (1.031, 1.036) |
| Adjusted for PM_2.5_ only | 1.025 (1.022, 1.029) |
| Adjusted for Ozone only | 1.028 (1.025, 1.031) |
| Adjusted for PM_2.5_ & Ozone | 1.024 (1.024, 1.028) |

^a^ Abbreviations: RR, Risk Ratio; CI, confidence interval

^b^ Adjusted for ozone and PM_2.5_

^c^ Low ozone= 33.95ppb (25^th^ percentile), high ozone = 52.56ppb (75^th^ percentile); low PM_2.5_ = 6.26 µg/m^3^ (25^th^ percentile), high PM_2.5_ = 13.06 µg/m^3^ (75^th^ percentile)

| **Table S7.** Association Between a 1°C Change in Maximum Temperature and Acute Kidney Failure (May – September 2008 –2012) | |
| --- | --- |
| Demographic Variables and Subgroups^a^ | Risk Ratio (95% CI) |
| Age, years^b^ |  |
| 4 or younger | 0.922 (0.836, 1.016) |
| 5 – 24 | 1.021 (0.993, 1.049) |
| 25 – 44 | 1.018 (1.006. 1.031) |
| 45 – 64 | 1.025 (1.019, 1.031) |
| 65 – 84 | 1.014 (1.010, 1.019) |
| 85 or older | 1.016 (1.009, 1.022) |
| Sex^b^ |  |
| Male | 1.019 (1.015, 1.023) |
| Female | 1.015 (1.011, 1.020) |
| Race/Ethnicity^b^ |  |
| White, Non-Hispanic | 1.018 (1.014, 1.023) |
| Black, Non-Hispanic | 1.015 (1.009, 1.022) |
| Hispanic | 1.019 (1.010, 1.029) |
| Other | 1.013 (1.003, 1.023) |
| Month^b^ |  |
| May | 1.012 (1.007, 1.017) |
| June | 1.025 (1.018, 1.031) |
| July | 1.022 (1.014, 1.031) |
| August | 1.023 (1.014, 1.032) |
| September | 1.013 (1.007, 1.020) |
|  |  |
| NYC only^b^ | 1.018 (1.013, 1.022) |
| Rest of NYS^b^ |  |
| Rural NYS | 1.016 (1.003, 1.030) |
| Urban NYS excluding NYC | 1.018 (1.014, 1.023) |
|  |  |
| Low Ozone/ Low PM_2.5_^c^ | 1.015 (1.011, 1.018) |
| High Ozone/ Low PM_2.5_^c^ | 1.020 (1.015, 1.025) |
| Low Ozone/ High PM_2.5_^c^ | 1.016 (1.012, 1.021) |
| High Ozone/ High PM_2.5_^c^ | 1.022 (1.018, 1.026) |
|  |  |
| Unadjusted | 1.021 (1.018, 1.024) |
| Adjusted for PM_2.5_ only | 1.018 (1.014, 1.021) |
| Adjusted for Ozone only | 1.019 (1.016, 1.022) |
| Adjusted for PM_2.5_ & Ozone | 1.017 (1.014, 1.021) |

^a^ Abbreviations: RR, Risk Ratio; CI, confidence interval

^b^ Adjusted for ozone and PM_2.5_

^c^ Low ozone= 33.95ppb (25^th^ percentile), high ozone = 52.56ppb (75^th^ percentile); low PM_2.5_ = 6.26 µg/m^3^ (25^th^ percentile), high PM_2.5_ = 13.06 µg/m^3^ (75^th^ percentile)

| Table S8. Association Between a 1°C Change in Maximum Temperature and Cardiovascular Diseases (May – September 2008 –2012) | |
| --- | --- |
| Demographic Variables and Subgroups^a^ | Risk Ratio (95% CI) |
| Age, years^b^ |  |
| 4 or younger | 1.012 (0.998, 1.027) |
| 5 – 24 | 0.999 (0.994, 1.004) |
| 25 – 44 | 1.003 (1.001, 1.005) |
| 45 – 64 | 1.001 (1.000, 1.002) |
| 65 – 84 | 1.001 (0.999, 1.002) |
| 85 or older | 1.000 (0.998, 1.002) |
| Sex^b^ |  |
| Male | 1.001 (1.000, 1.002) |
| Female | 1.001 (1.000, 1.002) |
| Race/Ethnicity^b^ |  |
| White, Non-Hispanic | 1.001 (1.000, 1.002) |
| Black, Non-Hispanic | 1.001 (0.999, 1.003) |
| Hispanic | 1.001 (0.999, 1.003) |
| Other | 1.001 (0.999, 1.003) |
| Month^b^ |  |
| May | 1.004 (1.003, 1.005) |
| June | 1.001 (1.000, 1.003) |
| July | 1.003 (1.001, 1.005) |
| August | 0.998 (0.996, 1.000) |
| September | 0.997 (0.996, 0.999) |
|  |  |
| NYC only^b^ | 1.001 (1.000, 1.002) |
| Rest of NYS^b^ |  |
| Rural NYS | 0.999 (0.997, 1.002) |
| Urban NYS excluding NYC | 1.001 (1.000, 1.002) |
|  |  |
| Low Ozone/ Low PM_2.5_^c^ | 1.001 (1.000, 1.001) |
| High Ozone/ Low PM_2.5_^c^ | 1.000 (0.999, 1.001) |
| Low Ozone/ High PM_2.5_^c^ | 1.002 (1.001, 1.003) |
| High Ozone/ High PM_2.5_^c^ | 1.001 (1.000, 1.002) |
|  |  |
| Unadjusted | 1.001 (1.000, 1.002) |
| Adjusted for PM_2.5_ only | 1.001 (1.000, 1.002) |
| Adjusted for Ozone only | 1.001 (1.000, 1.002) |
| Adjusted for PM_2.5_ & Ozone | 1.001 (1.000, 1.002) |

^a^ Abbreviations: RR, Risk Ratio; CI, confidence interval

^b^ Adjusted for ozone and PM_2.5_

^c^ Low ozone= 33.95ppb (25^th^ percentile), high ozone = 52.56ppb (75^th^ percentile); low PM_2.5_ = 6.26 µg/m^3^ (25^th^ percentile), high PM_2.5_ = 13.06 µg/m^3^ (75^th^ percentile)

**Figure S2.** Risk of heat stress and dehydration Hospitalizations/ ED visits by pre-existing comorbidities.


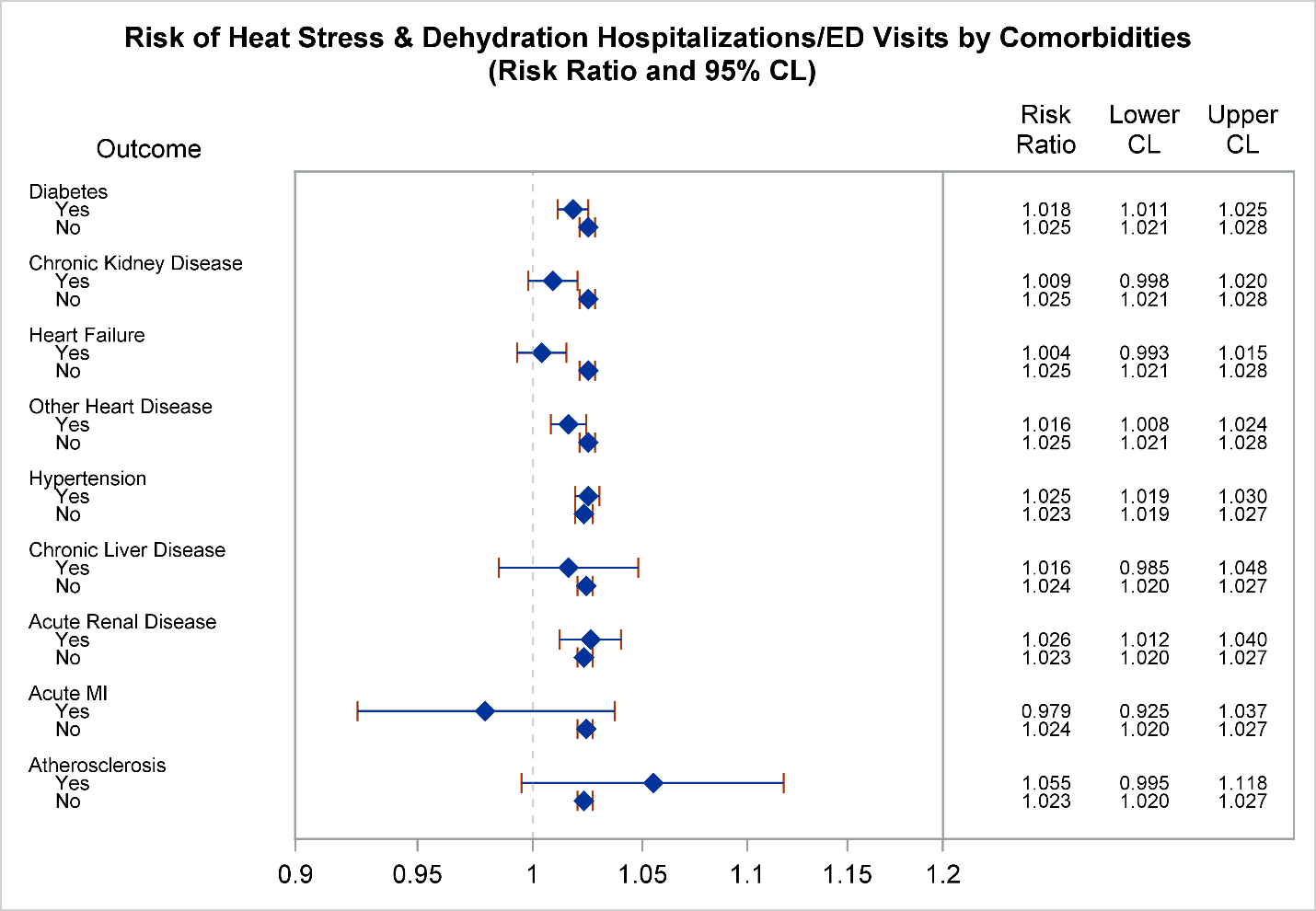


**Figure S3.** Map of Automated Surface Observing System (ASOS ) monitors in New York State


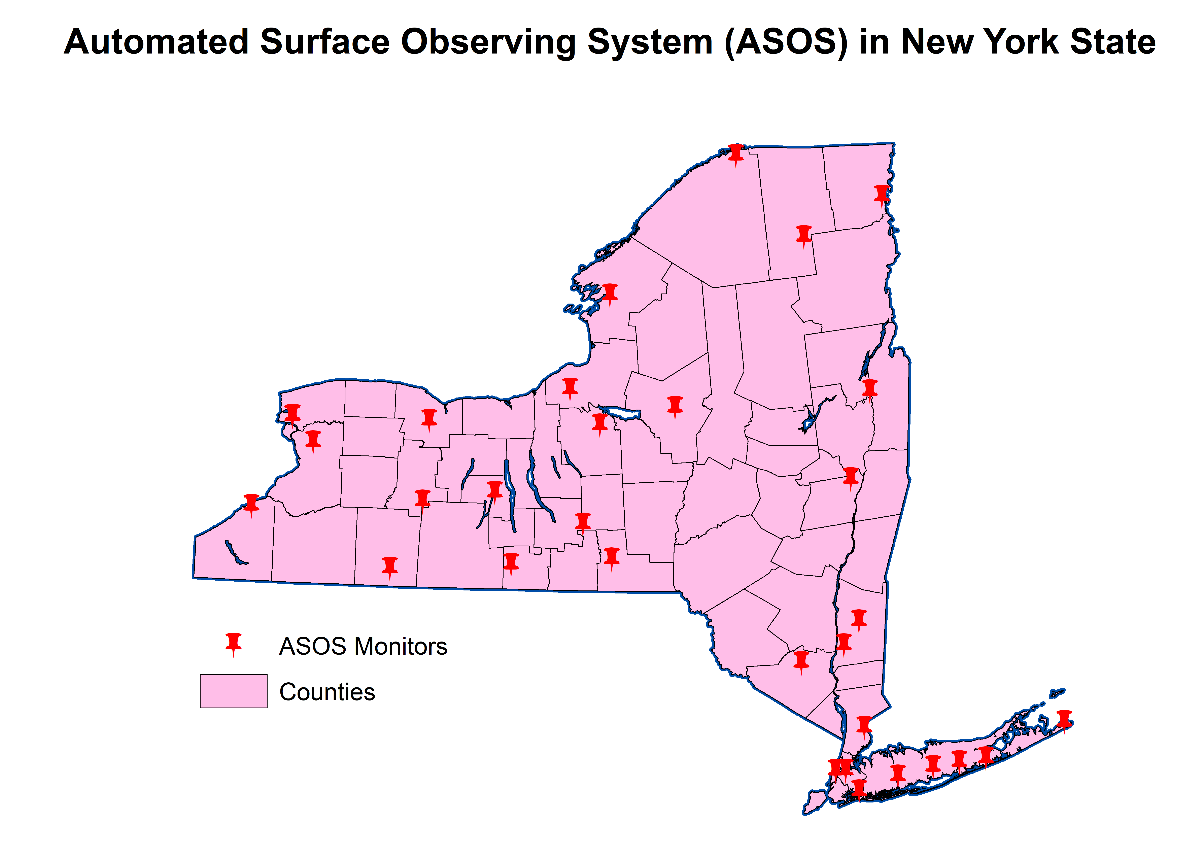


**Figure S4.** NLDAS maximum temperature (July 21, 2010) and heat stress cases (2008 – 2012) within 20-mile buffer radius of ASOS monitors


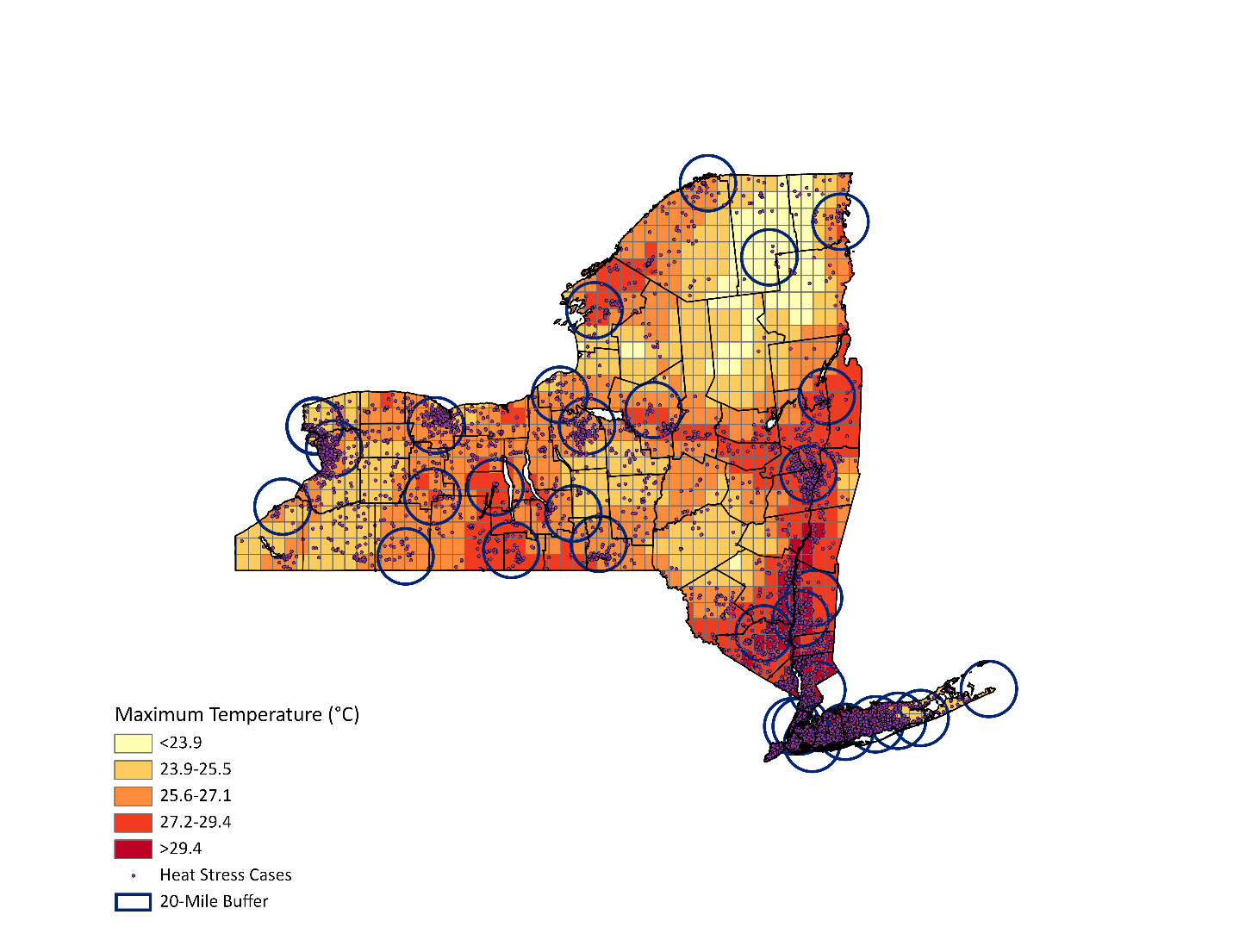


**Figure S5.** NLDAS maximum temperature (July 21, 2010) and heat stress cases (2008 – 2012) within 100-mile buffer radius of ASOS monitors


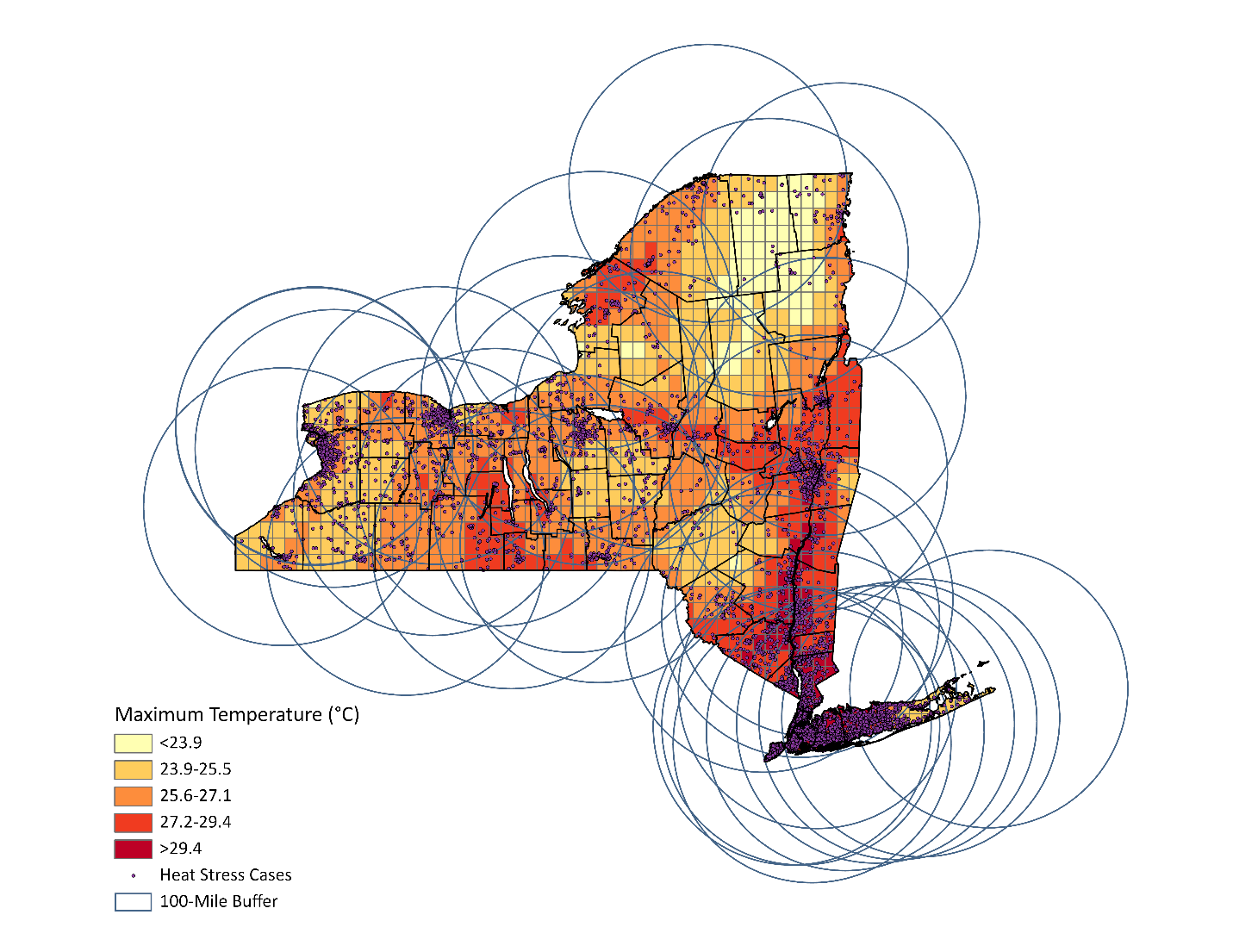


| **Table S9.** Spatial variations in risk estimates (NLDAS and ASOS monitor data (NYS)) | | | | | | | | |
| --- | --- | --- | --- | --- | --- | --- | --- | --- |
| NLDAS (12-km Grid)^a^ | | | Air Monitors (20-Mile Buffer)^a^ | | | Air Monitors (100-Mile Buffer)^a^ | | |
| Model | Risk Ratio | 95% CI | Model | Risk Ratio | 95% CI | Model | Risk Ratio | 95% CI |
| Lag 0 | 1.366 | 1.347, 1.386 | Lag 0 | 1.344 | 1.326, 1.362 | Lag 0 | 1.280 | 1.265, 1.295 |
| Lag 1 | 1.216 | 1.204, 1.229 | Lag 1 | 1.190 | 1.179, 1.202 | Lag 1 | 1.171 | 1.161, 1.182 |
| Lag 2 | 1.143 | 1.133, 1.153 | Lag 2 | 1.121 | 1.112, 1.131 | Lag 2 | 1.115 | 1.106, 1.125 |
| Lag 3 | 1.107 | 1.097, 1.117 | Lag 3 | 1.087 | 1.078, 1.096 | Lag 3 | 1.082 | 1.072, 1.091 |
| Lag 4 | 1.073 | 1.064, 1.082 | Lag 4 | 1.057 | 1.049, 1.066 | Lag 4 | 1.052 | 1.043, 1.061 |
| Lag 5 | 1.046 | 1.037, 1.054 | Lag 5 | 1.036 | 1.028, 1.044 | Lag 5 | 1.034 | 1.026, 1.043 |
| Lag 6 | 1.014 | 1.006, 1.023 | Lag 6 | 1.016 | 1.008, 1.024 | Lag 6 | 1.012 | 1.005, 1.020 |
| Lag 7 | 0.976 | 0.968, 0.984 | Lag 7 | 0.984 | 0.977, 0.992 | Lag 7 | 0.985 | 0.978, 0.993 |

^a^adjusted for PM_2.5_ and O_3_;

| **Table S10.** Spatial variations in risk estimates (NLDAS and ASOS monitor data (NYC only)) | | | | | | | | |
| --- | --- | --- | --- | --- | --- | --- | --- | --- |
| NLDAS (12-km Grid)^a^ | | | Air Monitors (20-Mile Buffer)^a^ | | | Air Monitors (100-Mile Buffer)^a^ | | |
| Model | Risk Ratio | 95% CI | Model | Risk Ratio | 95% CI | Model | Risk Ratio | 95% CI |
| Lag 0 | 1.371 | 1.338, 1.405 | Lag 0 | 1.376 | 1.345, 1.407 | Lag 0 | 1.321 | 1.294, 1.350 |
| Lag 1 | 1.237 | 1.215, 1.259 | Lag 1 | 1.235 | 1.215, 1.256 | Lag 1 | 1.197 | 1.178, 1.217 |
| Lag 2 | 1.173 | 1.155, 1.191 | Lag 2 | 1.158 | 1.142, 1.174 | Lag 2 | 1.149 | 1.131, 1.167 |
| Lag 3 | 1.135 | 1.117, 1.153 | Lag 3 | 1.105 | 1.090, 1.120 | Lag 3 | 1.096 | 1.078, 1.114 |
| Lag 4 | 1.083 | 1.067, 1.100 | Lag 4 | 1.059 | 1.045, 1.074 | Lag 4 | 1.051 | 1.035, 1.068 |
| Lag 5 | 1.055 | 1.040, 1.070 | Lag 5 | 1.038 | 1.025, 1.052 | Lag 5 | 1.033 | 1.018, 1.048 |
| Lag 6 | 1.031 | 1.017, 1.046 | Lag 6 | 1.031 | 1.018, 1.044 | Lag 6 | 1.021 | 1.006, 1.036 |
| Lag 7 | 0.983 | 0.969, 0.997 | Lag 7 | 0.987 | 0.974, 0.999 | Lag 7 | 0.987 | 0.973, 1.001 |

^a^adjusted for PM_2.5_ and O_3_;

| **Table S11.** Spatial variations in risk estimates (NLDAS and ASOS monitor data (Rest of NYS excluding NYC)) | | | | | | | | |
| --- | --- | --- | --- | --- | --- | --- | --- | --- |
| NLDAS (12-km Grid)^a^ | | | Air Monitors (20-Mile Buffer)^a^ | | | Air Monitors (100-Mile Buffer)^a^ | | |
| Model | Risk Ratio | 95% CI | Model | Risk Ratio | 95% CI | Model | Risk Ratio | 95% CI |
| Lag 0 | 1.363 | 1.339, 1.388 | Lag 0 | 1.326 | 1.304, 1.349 | Lag 0 | 1.263 | 1.245, 1.281 |
| Lag 1 | 1.204 | 1.189, 1.220 | Lag 1 | 1.165 | 1.151, 1.179 | Lag 1 | 1.159 | 1.146, 1.172 |
| Lag 2 | 1.127 | 1.115, 1.139 | Lag 2 | 1.101 | 1.090, 1.113 | Lag 2 | 1.101 | 1.090, 1.112 |
| Lag 3 | 1.094 | 1.083, 1.106 | Lag 3 | 1.078 | 1.067, 1.089 | Lag 3 | 1.075 | 1.065 1.086 |
| Lag 4 | 1.069 | 1.058, 1.080 | Lag 4 | 1.056 | 1.046, 1.067 | Lag 4 | 1.052 | 1.042, 1.063 |
| Lag 5 | 1.042 | 1.032, 1.053 | Lag 5 | 1.036 | 1.026, 1.046 | Lag 5 | 1.035 | 1.025, 1.044 |
| Lag 6 | 1.006 | 0.997, 1.016 | Lag 6 | 1.008 | 0.999, 1.018 | Lag 6 | 1.009 | 1.000, 1.018 |
| Lag 7 | 0.974 | 0.964, 0.983 | Lag 7 | 0.984 | 0.975, 0.993 | Lag 7 | 0.985 | 0.977, 0.994 |

^a^adjusted for PM_2.5_ and O_3_;

| **Table S12.** Spatial variations in risk estimates (NLDAS and ASOS monitor data (Rural NYS excluding NYC)) | | | | | | | | |
| --- | --- | --- | --- | --- | --- | --- | --- | --- |
| NLDAS (12-km Grid)^a^ | | | Air Monitors (20-Mile Buffer)^a^ | | | Air Monitors (100-Mile Buffer)^a^ | | |
| Model | Risk Ratio | 95% CI | Model | Risk Ratio | 95% CI | Model | Risk Ratio | 95% CI |
| Lag 0 | 1.373 | 1.302, 1.448 | Lag 0 | 1.355 | 1.263, 1.453 | Lag 0 | 1.307 | 1.249, 1.367 |
| Lag 1 | 1.226 | 1.179, 1.276 | Lag 1 | 1.195 | 1.139, 1.255 | Lag 1 | 1.177 | 1.138, 1.217 |
| Lag 2 | 1.157 | 1.120, 1.195 | Lag 2 | 1.125 | 1.080, 1.173 | Lag 2 | 1.147 | 1.112, 1.183 |
| Lag 3 | 1.111 | 1.078, 1.145 | Lag 3 | 1.104 | 1.060, 1.150 | Lag 3 | 1.096 | 1.065, 1.128 |
| Lag 4 | 1.075 | 1.045, 1.106 | Lag 4 | 1.071 | 1.031, 1.113 | Lag 4 | 1.073 | 1.044, 1.104 |
| Lag 5 | 1.039 | 1.012, 1.068 | Lag 5 | 1.043 | 1.005, 1.081 | Lag 5 | 1.043 | 1.016, 1.071 |
| Lag 6 | 0.997 | 0.970, 1.024 | Lag 6 | 1.004 | 0.970, 1.039 | Lag 6 | 1.010 | 0.985, 1.036 |
| Lag 7 | 0.972 | 0.947, 0.998 | Lag 7 | 0.987 | 0.954, 1.021 | Lag 7 | 0.981 | 0.957, 1.006 |

^a^adjusted for PM_2.5_ and O_3_;

**Table S13.** Spatial variations in risk estimates (NLDAS and ASOS monitor data (Urban NYS excluding NYC))

| NLDAS (12-km Grid)^a^ | | | Air Monitors (20-Mile Buffer)^a^ | | | Air Monitors (100-Mile Buffer)^a^ | | |
| --- | --- | --- | --- | --- | --- | --- | --- | --- |
| Model | Risk Ratio | 95% CI | Model | Risk Ratio | 95% CI | Model | Risk Ratio | 95% CI |
| Lag 0 | 1.362 | 1.337, 1.388 | Lag 0 | 1.324 | 1.301, 1.347 | Lag 0 | 1.258 | 1.240, 1.277 |
| Lag 1 | 1.201 | 1.185, 1.218 | Lag 1 | 1.163 | 1.148, 1.177 | Lag 1 | 1.156 | 1.142, 1.170 |
| Lag 2 | 1.123 | 1.110, 1.136 | Lag 2 | 1.099 | 1.087, 1.111 | Lag 2 | 1.095 | 1.083, 1.107 |
| Lag 3 | 1.092 | 1.080, 1.105 | Lag 3 | 1.076 | 1.064, 1.087 | Lag 3 | 1.073 | 1.061, 1.084 |
| Lag 4 | 1.068 | 1.056, 1.079 | Lag 4 | 1.055 | 1.044, 1.066 | Lag 4 | 1.049 | 1.038, 1.060 |
| Lag 5 | 1.043 | 1.032, 1.054 | Lag 5 | 1.035 | 1.025, 1.045 | Lag 5 | 1.034 | 1.023, 1.044 |
| Lag 6 | 1.008 | 0.997, 1.018 | Lag 6 | 1.008 | 0.998, 1.018 | Lag 6 | 1.009 | 0.999, 1.019 |
| Lag 7 | 0.974 | 0.964, 0.984 | Lag 7 | 0.983 | 0.974, 0.993 | Lag 7 | 0.986 | 0.977, 0.996 |

^a^adjusted for PM_2.5_ and O_3_;
